# A loss-of-function CACNA1A mutation causing benign paroxysmal torticollis of infancy

**DOI:** 10.1186/1129-2377-14-S1-P24

**Published:** 2013-02-21

**Authors:** M Vila-Pueyo, C Sintas, M Flotats, G Gené, X Elorza, JM Fernández-Fernández, B Cormand, A Macaya

**Affiliations:** 1 Pediatric Neurology Research Group, Vall d’Hebron Research Institute, Universitat Autònoma de Barcelona, Spain; 2 Departament of Genetics, Faculty of Biology, University of Barcelona, Spain; 3 Pediatric Neurology Service, Vall d’Hebron Hospital, Spain; 4 Laboratory of Molecular Physiology and Channelopathies, Department of Experimental and Health Sciences, Universitat Pompeu Fabra, Spain

## Introduction and background

Benign paroxysmal torticollis of infancy (BPTI) is a rare paroxysmal disorder characterized by recurrent episodes of head tilt and variable behavioural and autonomic changes, usually disappearing after age 2 years and often evolving into benign paroxysmal vertigo or common migraine. A few reports have linked BPTI to mutations in CACNA1A1.

## Patients and methods

A 2 year-old boy was referred with a history of recurrent episodes of torticollis starting at the age of 9 months and occurring twice per month ever since. During the episodes, which lasted from minutes to 2 hours and were relieved by sleep, the patient became irritable, unsteady and held onto his mother. After age 2 years the patient appeared drowsy and apathetic during the episodes. His psychomotor development and interictal examination are normal. Her 10-year-old female sister experienced similar attacks between ages 13 months and 3 years. They occurred monthly and lasted from 30 minutes to 24 hours and some reportedly associated upgaze deviation and severe global hypotonia. Carbamazepine did not help. No overt migraine attacks have developed. We performed direct sequencing of the 47 exons and flanking intronic regions of the CACNA1A gene in the nuclear family and a maternal aunt affected with epilepsy.

## Results

A heterozygous G-to-A transition in exon 12 (c.1597G>A) of CACNA1A, bringing about a p.Glu533Lys change, was found in both patients and their asymptomatic mother. The change involves a highly conserved glutamate on the S2 segment of domain II of the protein. Analysis of the mutant channel expressed in HEK 293 cells reveals that the mutation produces both a huge decrease in current density and a significant shift to higher voltages of the current activation curve that was accompanied by the alteration of activation kinetics. A previous report has shown cosegregation of p.Glu533Lys with familial episodic ataxia type-2 (EA-2)2.

## Conclusion

This is the first report of a childhood periodic syndrome being caused by a loss-of-function CACNA1A mutation.
